# Epidemiological data on the acquisition of carbapenem-resistant *Enterobacterales* through weekly rectal swabs in non-critically ill patients undergoing antimicrobial therapy: a short-term surveillance study

**DOI:** 10.1017/ash.2025.169

**Published:** 2025-05-13

**Authors:** Naruemit Sayabovorn, Naruemon Maknakhon, Naratchaphan Pati, Teerawit Tangkoskul, Anupop Jitmuang

**Affiliations:** 1 Division of Ambulatory Medicine, Department of Medicine, Faculty of Medicine Siriraj Hospital, Mahidol University, Bangkok, Thailand; 2 Division of Infectious Diseases and Tropical Medicine, Department of Medicine, Faculty of Medicine Siriraj Hospital, Mahidol University, Bangkok, Thailand

## Abstract

**Objective:**

To evaluate the connection between non-critically ill hospitalized patients and the acquisition of carbapenem-resistant *Enterobacterales* (CRE).

**Design:**

An observational prospective cohort study from January 2018 to December 2019.

**Setting:**

A single tertiary referral center.

**Participants:**

Non-critically ill subjects admitted to general medical wards who received antimicrobial therapy <48 h.

**Methods:**

Rectal swab cultures at admission and weekly for CRE surveillance. CRE isolates were confirmed using carbapenem disk diffusion susceptibility and genotypic carbapenemase testing. Clinical characteristics and outcomes were also evaluated.

**Results:**

Of 110 subjects, 66.4% were women, the mean age was 67 years, and 336 bacterial isolates were detected from rectal swab cultures. 55 (16.4%) isolates from 25 subjects exhibited phenotypic resistance to carbapenem. *Klebsiella pneumoniae* (50.9%) and *Escherichia coli* (30.9%) were common CRE, harboring New Delhi metallo-beta-lactamase (NDM) (50.9%), oxacillinase-48 (OXA-48) (12.7%), and co-NDM/OXA-48 (20.0%). Subjects with acquired CRE had higher APACHE II scores (*P* = 0.030), received piperacillin-tazobactam (*P* = 0.004), underwent prolonged antimicrobial therapy (*P* = 0.009), and experienced longer hospital stays (*P* = 0.001) compared to CRE-negative subjects. None of the CRE-positive subjects developed an acquired infection.

**Conclusions:**

Acquired CRE colonization is prevalent among non-critically ill patients. Factors such as disease severity, the type and duration of antimicrobial therapy, and the length of hospital stays may increase the risk of CRE acquisition in non-critically ill populations.

## Introduction

Carbapenem-resistant *Enterobacterales* (CRE) is an emerging multidrug-resistant organism (MDRO) that is becoming more widespread in healthcare facilities. The emergence of this MDRO leads to the restriction of antimicrobial treatment options.^1^ Therefore, the mortality rate is increasing among hospitalized patients with CRE infections. In Thailand, the rate of CRE, tested by isolates resistant to one of the carbapenem antibiotics, has changed rapidly in the past ten years.^2,3^ Several factors, such as prolonged hospitalization, critical illness, mechanical ventilatory support, long-term use of antimicrobial therapy, particularly carbapenems, and previous colonization, are risk factors for acquiring CRE infection in critically ill populations.^4–6^ In addition, the selective pressure from the use of broad-spectrum antimicrobials, especially in critically ill populations, can disrupt gut microbiota, resulting in acquired CRE colonization.^7^ Thus, CRE carriage and reservoir surveillance, and infection control measures to prevent further spreading are mainly focused on high-risk or critically ill patients.^8^

However, CRE acquisition in non-critically ill populations must be recognized and more concerned. In addition, the relationship between the use of antimicrobial therapy in non-critically ill hospitalized populations and the acquisition of CRE infection or colonization during a hospital stay remains unclear. The study aimed to assess whether early antimicrobial therapy administered within 48 h of hospitalization increases the risk of CRE acquisition in these populations.

## Methods

### Study design

We conducted an observational prospective cohort study to assess the prevalence of CRE acquisition in subjects admitted to general medicine wards who received antimicrobial therapy after hospitalization at Siriraj Hospital from January 2018 to December 2019. To assess the impact of early antimicrobial therapy on the acquisition of CRE, we included only adult subjects aged >18 years who received antimicrobial therapy within 48 h prior to enrollment. We excluded subjects who had received antimicrobial therapy within the past 30 days, had absolute neutrophil counts <1000 cells/mm^3^, or did not consent to participate. The study received approval from the Institutional Review Board of the Faculty of Medicine at Siriraj Hospital, Mahidol University (Si 530/2017). Informed consent was obtained from all subjects involved in the study.

### Study procedures

After subject enrollment, we performed an initial rectal culture swab for CRE surveillance, followed by the rectal swab every 5–7 days (or weekly) during the subject’s hospitalization. The rectal culture swab was placed in a single tube with Cary Blair transport medium and sent to the Infectious Diseases and Tropical Medicine Research Laboratory for CRE testing according to the Centers for Disease Control and Prevention protocol.^9^ Briefly, the swab was inoculated in 5 mL of trypticase soy broth (TSB) with one 10-μg of ertapenem (ETP) for incubation overnight at 35 °C. After overnight incubation, the incubated TSB was mixed by vortexing and pipetted 100 μL to inoculate onto a MacConkey agar plate for subculture. Lactose-fermenting Gram-Negative colonies, including ≥1 colony morphology, were selected to be subcultured in TSB and then incubated for 4 h at 35 °C for isolation. Conventional biochemical tests were used to identify each isolate that grew into TSB, and the isolate was tested for carbapenem susceptibility by ETP, meropenem (MER), and imipenem (IMI) disk diffusion (DD) susceptibility testing according to the Clinical and Laboratory Standards Institute (CLSI) guideline.^10^ CRE isolate was defined as *Enterobacterales* with carbapenem susceptibility testing showing resistance to at least one of the carbapenem agents according to the CLSI criteria.^10–12^

*K*. *pneumoniae* carbapenemase (KPC), New Delhi metallo-beta-lactamase (NDM), imipenemase (IMP), Verona integron-encoded metallo-beta-lactamase (VIM), and oxacillinase-48 (OXA-48) are predominant carbapenemases widely distributed among carbapenemase-producing *Enterobacterales*.^13^ Therefore, we conducted in-house genotypic tests to detect KPC, NDM, IMP, VIM, and OXA-48 carbapenemases in phenotypic CRE isolates as the primary carbapenemase targets available for testing at our institute. All phenotypic CRE strains were assessed to determine their genotypic carbapenemase. Two to three isolate colonies were inoculated in TSB and incubated overnight. The isolates were suspended in distilled water and homogenized by vortexing. The precipitated isolate particles were suspended in distilled water and then heated in a water bath at 95 °C for 10 min, and then the suspension was cooled. The suspension was centrifuged for 3 min at 10,000 rpm, and the 100 μL supernatant was prepared for polymerase chain reaction (PCR). One microliter of total DNA was subjected to singleplex PCR in a 20-μL reaction mixture. The forward (F) and reverse (R) primers used for carbapenemase gene testing were those previously described by Poirel et al. and Ellington et al. as the following: for *bla*_NDM_, 5′-GGT TTG GCG ATC TGG TTT TC-3′ (F) and 5′-CGG AAT GGC TCA TCA CGA TC-3′ (R); for *bla*_OXA-48_, 5′-GCG TGG TTAAGG ATG AAC AC-3′ (F) and 5′-CAT CAA GTT CAA CCC AAC CG-3′ (R); for *bla*_KPC_, 5′-CGT CTA GTT CTG CTG TCT TG-3′ (F) and 5′- CTT GTC ATC CTT GTT AGG CG-3′ (R); for *bla*_VIM_, 5′-GAT GGT GTT TGG TCG CAT AT-3′ (F) and 5′-CGA ATG CGC AGC ACC AG-3′ (R); and for *bla*_IMP_, 5′-GGA ATA GAG TGG CTT AAT TCT C-3′ (F) and 5′- CCA AAC CAC TAC GTT ATC T-3′ (R).^14,15^ The mixture for amplification reactions contained 1 μL of DNA template, 2 μL of dNTP Mix (2.5 mM each), 1 μL of each F and R primers, 2 μL of 10x *i*-Taq plus PCR buffer (Thermo Fisher Scientific, Inc., Waltham, MA, USA), and 0.2 μL of *i*-Taq plus DNA polymerase (Thermo Fisher Scientific, Inc., Waltham, MA, USA), those were mixed with sterile distilled water to generate the 20-μL PCR mix. The cycling conditions comprised an initial DNA denaturation at 94 °C for 5 min, followed by 35 cycles of 94 °C for 20 s (s), 50 °C for 20 s, and 72 °C for 30 s. Subsequently, the final elongation of the template was done at 72 °C for 5 min to complete the amplification. The PCR products were analyzed by 1% agarose gel electrophoresis at 100 volts for 30 min in 0.5x Tris-borate-EDTA (TBE) buffer.

The weekly rectal culture swab was discontinued if one of the following criteria was met: The subject was dead, discharged, or transferred to other healthcare units, and the subject subsequently denied participating in the study. Clinical data were collected from patients’ medical and electronic records. Clinical characteristics, management, and treatment outcomes were also evaluated. According to our hospital policy, infection prevention and control (IPC) measures have been implemented for all CRE-positive patients from any site of sample cultures. These measures include strict contact precautions and hand hygiene, wearing disposable gowns and latex gloves, using individualized medical devices, and reinforcement of environmental cleaning in all affected areas.

### Definitions

We classified the results of the rectal swab culture into positive and negative CRE cultures. After receiving antimicrobial therapy, the CRE-positive culture was defined as CRE isolates phenotypically identified from the rectal swab culture. If the initial rectal swab culture discovered CRE isolates while antimicrobial therapy was administered within 48 h at enrollment, we consider that this finding could not be related to the emerging CRE after antimicrobial therapy. The CRE-negative culture was defined as rectal swab cultures that did not discover CRE isolates throughout the study period.

### Statistical analysis

The overall prevalence of CRE in Asian countries was approximately10.9%.^6^ According to the proportion of one group method, an estimated error of 5%, and a 95% confidence interval, the calculated sample size was 150 patients. Statistical analysis was analyzed using SPSS version 28.0 (IBM Corp, Armonk, NY, USA). Variables and values were expressed as mean ± standard deviation or median (interquartile range, IQR) for continuous variables and as frequencies (percentage) for categorical variables. We compared the continuous variable using the Student’s two-tailed *t*-test or the Mann–Whitney *U* test for nonparametric testing. The chi-square and Fisher’s exact tests were used to analyze differences between categorical variables. Univariate and multivariate logistic regression models investigated associations between clinical factors and CRE-positive rectal swab culture. The statistically significant result was considered as *P* < 0.05.

## Results

**Figure 1.**
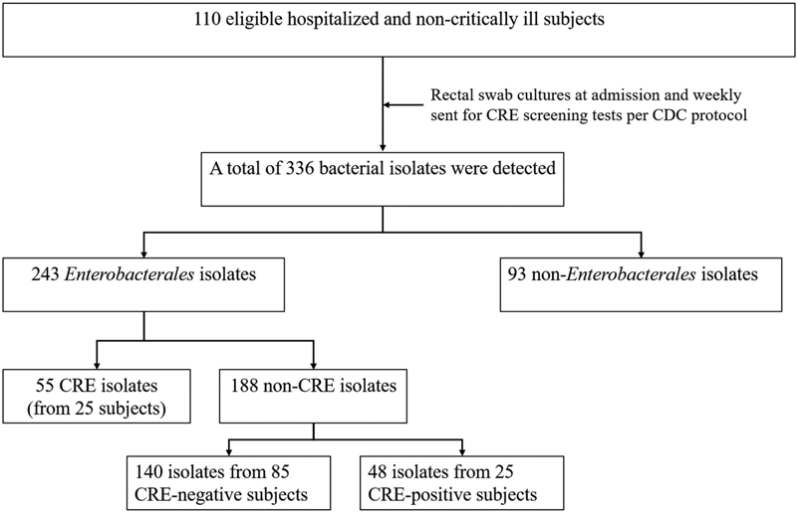
Study workflow.

One hundred and ten non-critically ill hospitalized subjects were eligible for a rectal culture swab to monitor for CRE isolates after receiving systemic antimicrobial therapy for up to 48 h. The sample size did not reach 150 calculated subjects, as we needed more resources and laboratory equipment to continue the microbiological study throughout the study. Of 110 subjects, 336 bacterial isolates were isolated from the rectal swab cultures. Fifty-five *Enterobacterales* isolates from 25 subjects (22.7%) were resistant to carbapenems, while 188 *Enterobacterales* were non-carbapenem-resistant strains (Figure 1). Therefore, the prevalence of CRE acquisition among all isolates during the study period was 16.4%. Additionally, 85 subjects found only non-carbapenem-resistant *Enterobacterales* throughout the study period.

Table 1 demonstrates the clinical characteristics, severity, management, and outcomes of the subjects between the CRE negative and positive culture groups. Subjects with CRE-positive rectal swab cultures had significantly higher acute physiology and chronic health evaluation (APACHE) II scores (*P* = 0.030) and sequential organ failure assessment (SOFA) scores (*P* = 0.214) than those with CRE-negative cultures. Common sites of infection were lower respiratory tract infection (40.0%), followed by gastrointestinal tract infection (19.1%), and urinary tract infection (17.3%). Gastrointestinal tract infection and primary bacteremia were seemingly different infectious foci between the two groups. The most common antimicrobial agent administered to treat infection episodes was ceftriaxone (70.0%), followed by piperacillin-tazobactam (34.5%) and azithromycin (28.2%). Among antimicrobial agents, piperacillin-tazobactam was administered more frequently in the CRE-positive group (60.0%) than in the CRE-negative group (27.0%) (*P* = 0.004). Furthermore, the duration of antimicrobial therapy was significantly longer in the CRE-positive group (*P* = 0.009). Most subjects (77.3%) had retained medical devices, with 24.5% receiving blood and its components transfusions. The study shows that CRE-positive subjects received significant blood transfusion therapy (*P* = 0.016) and were more likely to retain medical devices (*P*= 0.057) compared to CRE-negative subjects. The overall treatment outcomes for individuals with positive and negative CRE tests were similar. Only 12 (10.9%) participants with a noncritical illness had fatal outcomes, while 97 (88.2%) recovered. However, the median length of hospital stay in the CRE-positive group was significantly longer than in the CRE-negative group (17.0 vs. 8.0 days, *P* = 0.001). Similarly, the results of the “time from admission to rectal swab outcomes” explain this finding. Among all 25 CRE-positive subjects, CRE isolates were not identified during the initial rectal swab surveillance but were discovered later, averaging 8 (4.5, 14.0) days after hospitalization (*P* = 0.024). All CRE-positive cultures (25) exhibited sustained CRE colonization throughout their hospital stays, and none developed an acquired infection.

**Table 1.** Clinical characteristics, severity and site of infections, antimicrobial therapy, management, and results of study subjects between CRE negative and positive rectal swab culture groups

Parameter	CRE-negative rectal swab cultures (n = 85)	CRE-positive rectal swab cultures (n = 25)	*p*-value
Gender, n (%)			
Female	55 (64.7%)	18 (72.0%)	0.632
Age (years)			
Mean 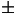 S.D.	67.7 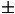 16.3	67.9 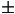 18.2	0.958
Body weight (kg)			
Mean ± S.D.	54.0 ± 12.5	58.1 ± 13.4	0.628
Physical status, n (%)			0.292
Capable of self-care	57 (67.1%)	13 (52.0%)	
Living in partial independence status	15 (17.6%)	5 (20.0%)	
Living in dependence status	13 (15.3%)	7 (28.0%)	
Coexisting medical condition, n (%)			
Hypertension	49 (57.6%)	10 (40.0%)	0.171
Diabetes mellitus	33 (38.8%)	3 (12.0%)	0.015
Dyslipidemia	38 (44.7%)	5 (20.0%)	0.035
Cerebrovascular disease	15 (17.6%)	7 (28.0%)	0.266
Chronic kidney disease	16 (18.8%)	7 (28.0%)	0.401
ESRD with long-term hemodialysis	5 (5.9%)	0 (0.0%)	0.586
Coronary artery disease	19 (22.4%)	2 (8.0%)	0.150
Arrhythmia	7 (8.2%)	4 (16.0%)	0.267
Chronic liver disease	10 (11.8%)	0 (0.0%)	0.113
HIV infection	3 (3.5%)	1 (4.0%)	1.000
Asthma or COPD	5 (5.9%)	1 (4.0%)	1.000
Others	66 (77.6%)	17 (68.0%)	0.428
Immunodeficiency, n (%)	13 (15.3%)	5 (20.0%)	0.552
Charlson comorbidity index			
Mean 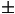 S.D.	5.9 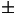 3.0	5.4 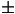 2.5	0.524
APACHE II score at admission,			
Mean 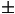 S.D.	15.6 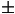 7.0	19.1 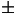 6.7	0.030
SOFA score at admission,			
Median (IQR)	2.0 (1.0, 4.5)	3.0 (2.0, 5.0)	0.214
**Site of infection, n (%)**			0.044
Lower respiratory tract	34 (40.0%)	10 (40.0%)	
Gastrointestinal tract	19 (22.4%)	2 (8.0%)	
Cardiovascular system	2 (2.4%)	0 (0.0%)	
Urinary tract infection	15 (17.6%)	4 (16.0%)	
Skin and soft tissue infections	3 (3.5%)	0 (0.0%)	
Central nervous system	0 (0.0%)	2 (8.0%)	
Bone and joint	1 (1.2%)	0 (0.0%)	
Primary bacteremia	11 (12.9%)	7 (28.0%)	
**Type of antimicrobial therapy, n (%)**			
Ceftriaxone	59 (69.4%)	18 (72.0%)	1.000
Amoxycillin-clavulanate	8 (9.4%)	4 (16.0%)	0.464
Piperacillin-tazobactam	23 (27.0%)	15 (60.0%)	0.004
Meropenem	14 (16.5%)	2 (8.0%)	0.518
Imipenem-cilastatin	2 (2.4%)	0 (0.0%)	1.000
Ertapenem	0 (0.0%)	1 (4.0%)	0.227
Levofloxacin	7 (8.2%)	2 (8.0%)	1.000
Co-trimoxazole	3 (3.5%)	3 (12.0%)	0.129
Metronidazole	7 (8.2%)	0 (0.0%)	0.347
Azithromycin	24 (28.2%)	7 (28.0%)	1.000
Vancomycin	6 (7.1%)	2 (8.0%)	1.000
Colistin	1 (1.2%)	2 (8.0%)	0.129
Others	17 (20.0%)	9 (36.0%)	0.113
**Number of antimicrobial uses**			0.060
Median (IQR)	2 (1, 2)	2 (1, 3)	
**Duration of antimicrobial therapy (days)**			0.009
Median (IQR)	7 (5, 11)	10 (7, 25)	
Medical devices used^a^, n (%)	62 (72.9%)	23 (92.0%)	0.057
Receiving blood and its component transfusion, n (%)	16 (18.8%)	11 (44.0%)	0.016
Undergoing surgical intervention, n (%)	31 (36.5%)	10 (40.0%)	0.816
**Treatment outcomes, n (%)**			0.569
Recovery	76 (89.4%)	21 (84.0%)	
Not improved	1 (1.2%)	0 (0.0%)	
In-hospital mortality	8 (9.4%)	4 (16.0%)	
**Length of hospital stay (days)**			
Median (IQR)	8.0 (5.0, 15.0)	17.0 (9.5, 39.0)	0.001
**Interval from admission to rectal swab outcomes** ^**b**^ **(days)**			
Median (IQR)	5.0 (1.0, 8.5)	8.0 (4.5, 14.0)	0.024
**Number of weekly rectal swabs per case (time)**			
Median (IQR)	1 (1, 2)	2 (1.5, 2)	0.024
**In-hospital outcomes of CRE-positive rectal swab culture, n (%)**			na
Sustained colonization	–	25 (100%)	
Subsequent CRE infection	–	0 (0.0%)	

Abbreviations: (APACHE), acute physiology and chronic health evaluation; (COPD), chronic obstructive pulmonary disease; (CRE), Carbapenem-resistant *Enterobacterales*; (ESRD), end-stage renal disease; (HIV), human immunodeficiency virus; (na), not applicable; (SOFA), sequential organ failure assessment.

a Medical devices used, such as endotracheal intubation, nasogastric tubing, urinary catheterization, and central venous catheterization.

b In the CRE-negative group, the intervals were defined as the time between the hospital admission date and the final date of the rectal culture swab. In the CRE-positive group, the intervals were defined as the time between the hospital admission date and the first date of the CRE-positive rectal swab culture.

Additionally, the multivariate logistic regression analysis indicates that no clinical parameter was an independent factor significantly associated with acquiring CRE detected through rectal swab culture (Table 2).

**Table 2.** Multivariate logistic regression analysis of factors associated with CRE-positive rectal swab culture

Variables	Adjusted odd ratios	95% CI	*p-value*
Male sex	2.23	0.59, 8.46	0.238
Body weight (increased by 1 kg)	1.03	0.98, 1.08	0.222
Having any coexisting medical condition	0.24	0.04, 1.39	0.111
Charlson comorbidity index (increased by 1 score)	0.87	0.68, 1.13	0.296
APACHE II score (increased by 1 score)	1.07	0.98, 1.17	0.123
Retaining any medical devices	2.27	0.39, 13.14	0.359
Receiving blood and its component transfusion	3.14	0.89, 11.10	0.075
Administration of piperacillin-tazobactam	2.78	0.90, 8.65	0.077
Administration of carbapenems	0.16	0.03, 1.06	0.057
Interval from admission to rectal swab outcomes (increased by 1 day)	1.03	0.97, 1.09	0.358

Abbreviations: (APACHE), acute physiology and chronic health evaluation; (kg), kilogram.

### Results of susceptibility testing for carbapenem disk diffusion and genotypic carbapenemase of all carbapenem-resistant Enterobacterales isolates

55 CRE isolates include *K. pneumoniae* (50.9%), followed by *E. coli* (30.9%), *Enterobacter* spp. (10.9%) and *Citrobacter* spp. (7.3%). The in-house carbapenemase gene assays show that NDM (50.9%), OXA-48 (12.7%), and coexistent NDM and OXA-48 (20.0%) are common carbapenemases found among CRE isolates (Table 3). Most CRE isolates that conferred OXA-48-type carbapenemase displayed more variable susceptibility patterns to carbapenem disk diffusion. Meanwhile, most CRE isolates with NDM-type carbapenemase showed greater resistance to all or at least two carbapenem agents. Only 5 CRE isolates—four *Enterobacter* spp. and one *E. coli*—exhibited phenotypic resistance to at least one of the carbapenems; however, targeted carbapenemase genes were not detected. Regrettably, two carbapenem-resistant strains of *K. pneumoniae* were lost during the study, leading to the unavailability of genotypic testing data.

**Table 3.** Results of carbapenem disk diffusion susceptibility testing and genotypic carbapenemase testing of all carbapenem-resistant *Enterobacterales* isolates

No.	Type of isolate	Inhibition zone diameter (millimeter) and interpretation of carbapenem DD susceptibility testing^a^						Result of in-house genotypic carbapenemase testing				
		ETP		IMI		MER		IMP	NDM	OXA-48	KPC	VIM
1	*Enterobacter* spp.	7	R	17	R	13	R	–	–	–	–	–
2	*Enterobacter* spp.	13	R	24	S	23	S	–	–	–	–	–
3	*Enterobacter* spp.	14	R	24	S	23	S	–	–	–	–	–
4	*K. pneumoniae* ^b^	6	R	6	R	6	R	na	na	na	na	na
5	*K. pneumoniae* ^b^	6	R	6	R	6	R	na	na	na	na	na
6	*Enterobacter* spp.	21	I	16	R	23	S	–	–	–	–	–
7	*K. pneumoniae*	11	R	25	S	19	R	–	+	–	–	–
8	*K. pneumoniae*	8	R	24	S	16	R	–	+	–	–	–
9	*E. coli*	16	R	28	S	23	S	–	–	–	–	–
10	*K. pneumoniae*	6	R	16	R	11	R	–	+	+	–	–
11	*E. coli*	12	R	19	R	15	R	–	+	+	–	–
12	*Citrobacter* spp.	15	R	24	S	19	R	+	–	–	–	–
13	*Citrobacter* spp.	17	R	24	S	24	S	–	–	+	–	–
14	*Citrobacter* spp.	14	R	24	S	22	I	–	–	+	–	–
15	*Citrobacter* spp.	16	R	25	S	24	S	–	–	+	–	–
16	*K. pneumoniae*	16	R	29	S	23	S	–	+	–	–	–
17	*K. pneumoniae*	13	R	28	S	22	I	–	+	–	–	–
18	*K. pneumoniae*	6	R	23	S	15	R	–	+	–	–	–
19	*K. pneumoniae*	11	R	18	R	13	R	–	+	–	–	–
20	*E. coli*	15	R	19	R	16	R	–	+	–	–	–
21	*K. pneumoniae*	16	R	27	S	22	I	–	+	–	–	–
22	*K. pneumoniae*	8	R	22	I	15	R	–	+	–	–	–
23	*K. pneumoniae*	8	R	21	I	16	R	–	+	–	–	–
24	*K. pneumoniae*	14	R	26	S	21	I	–	+	–	–	–
25	*E. coli*	7	R	14	R	12	R	–	+	–	–	–
26	*E. coli*	6	R	12	R	12	R	–	+	–	–	–
27	*K. pneumoniae*	11	R	16	R	14	R	–	+	–	–	–
28	*E. coli*	6	R	11	R	11	R	–	+	–	–	–
29	*E. coli*	6	R	13	R	12	R	–	+	–	–	–
30	*E. coli*	7	R	15	R	13	R	–	+	–	–	–
31	*E. coli*	7	R	12	R	11	R	–	+	–	–	–
32	*Enterobacter* spp.	15	R	19	R	17	R	+	–	–	–	–
33	*K. pneumoniae*	8	R	20	I	14	R	–	+	+	–	–
34	*K. pneumoniae*	9	R	18	R	14	R	–	+	+	–	–
35	*E. coli*	18	R	26	S	26	S	–	–	+	–	–
36	*K. pneumoniae*	15	R	19	R	16	R	–	+	–	–	–
37	*Enterobacter* spp.	10	R	17	R	15	R	–	+	–	–	–
38	*E. coli*	15	R	16	R	16	R	–	+	–	–	–
39	*E. coli*	17	R	24	S	24	S	–	+	–	–	–
40	*E. coli*	6	R	15	R	8	R	–	–	+	–	–
41	*E. coli*	17	R	25	S	22	I	–	–	+	–	–
42	*E. coli*	6	R	18	R	9	R	–	+	–	–	–
43	*K. pneumoniae*	9	R	27	S	17	R	–	+	–	–	–
44	*K. pneumoniae*	8	R	27	S	16	R	–	+	–	–	–
45	*E. coli*	6	R	16	R	6	R	–	+	–	–	–
46	*E. coli*	9	R	16	R	13	R	–	+	–	–	–
47	*K. pneumoniae*	17	R	24	S	23	S	–	+	+	–	–
48	*K. pneumoniae*	14	R	24	S	20	I	–	–	+	–	–
49	*K. pneumoniae*	18	R	24	S	24	S	–	+	+	–	–
50	*K. pneumoniae*	18	R	25	S	24	S	–	+	+	–	–
51	*K. pneumoniae*	16	R	20	I	19	R	–	+	+	–	–
52	*K. pneumoniae*	17	R	21	I	19	R	–	+	+	–	–
53	*K. pneumoniae*	9	R	16	R	13	R	–	+	+	–	–
54	*K. pneumoniae*	6	R	13	R	8	R	–	+	+	–	–
55	*K. pneumoniae*	10	R	18	R	15	R	–	+	–	–	–

-, genotypic testing negative; +, genotypic testing positive.

Abbreviations: (ETP), ertapenem; **(**IMI), imipenem; **(**IMP), imipenemase; (I), intermediate; (KPC), *K. pneumoniae* carbapenemase; (MER), meropenem; (na), not available; (NDM), New Delhi metallo-beta-lactamase; (OXA-48), oxacillinase-48; (R), resistant; (S), susceptible; (VIM), Verona integron-encoded metallo-beta-lactamase

a Carbapenem susceptibility was tested using the disk diffusion (DD) method and interpretative criteria according to the CLSI guideline 2018.

b Isolates No. 4 and 5 were misplaced during the study, resulting in unavailable genotypic data.

## Discussion

The epidemiology of CRE acquisition, assessed through weekly rectal swab cultures in non-critically ill subjects undergoing antimicrobial therapy, was 16.4%. Among the CRE isolates, *K. pneumoniae* was the most common species. Our findings are consistent with data from the National Antimicrobial Resistance Surveillance, Thailand (NARST) 2000–2021,^2^ which indicated that the rate of carbapenem-resistant *K. pneumoniae* has risen from 0.4% to 17.9% over the past 20 years.^2^

A prior study reported an indirect correlation between overall antimicrobial consumption and the prevalence of CRE.^16^ Our study indicates that antimicrobial therapy can significantly affect the increasing prevalence of CRE in hospitalized, non-critically ill patients. However, this study provides a short-term surveillance of CRE rectal swab cultures. Consequently, we do not know how long patients with CRE-positive rectal swab cultures were colonized at the time of antimicrobial discontinuation and their discharge home. A long-term surveillance study revealed that the time from CRE-positive to CRE-negative rectal swab cultures after discharge varied, ranging from 20 to 188 days, while 22.0% had persistent CRE carriage.^17^ Furthermore, the prevalence of CRE varies based on the patient’s setting and severity; for example, the prevalence of CRE acquisition in critically ill patients was significantly higher compared to our study.^18,19^

When comparing clinical characteristics and outcomes, patients with CRE-positive cultures had significantly higher APACHE II scores than those with CRE-negative cultures. Increased dependence status and immunodeficiency, including bacteremia, can result in greater severity and higher APACHE II scores in the CRE-positive group compared to the CRE-negative group. The APACHE II score is a significant risk factor associated with acquired carbapenem-resistant Gram-negative bacteria infections.^20^ We also identified a significant difference in the type of antimicrobial administration between the two groups. Before the acquisition of CRE, piperacillin-tazobactam was the only agent used more significantly in the CRE-positive group. The broad-spectrum activity of this agent may cause dysbiosis and exert selective pressure on gut microbiota, causing emergence of CRE.^7^ Previous studies indicate that exposure to certain antimicrobial agents, such as cephalosporins, beta-lactam/beta-lactamase inhibitor combinations, carbapenems, and fluoroquinolones, increases the risk of acquiring CRE in hospitalized patients.^21–24^ Carbapenems are the antimicrobial agents most strongly linked to the acquisition of CRE.^17,18,22^ However, this study did not establish such a link, as carbapenems were prescribed to only a few study populations. Furthermore, our hospital has implemented an antibiotic stewardship program to restrict carbapenem use in selected cases since 2007.^25^

A larger proportion of patients with CRE-positive cultures received medical devices (*P* = 0.057). There is a correlation between CRE-positive cases and the use of medical devices or procedures, such as mechanical ventilation, urinary catheterization, and central venous catheterization reported.^26,27^ Moreover, patients with CRE-positive cultures received significantly more blood and component transfusions than those with CRE-negative cultures, which increases the risk of acquiring CRE with the number of transfusions received.^28^ This study also showed that patients with positive CRE tests had a median onset of CRE acquisition of 8 days. Torres-Gonzalez et al. found that the median length of stay before detecting CRE isolates in fecal samples was 8 days, similar to our findings.^5^ Patients with a more extended hospital stay have a higher risk of CRE infection.^29^ Surprisingly, although the CRE-positive group had a longer hospital stay (median of 17 days), none developed an acquired CRE infection. According to our study, multivariate analysis indicates that no clinical parameters are independent factors associated with CRE-positive cultures. The small sample size and the effects of multicollinearity may have contributed to this finding. Moreover, several factors, such as sharing space with a known CRE carrier, colonization pressure, environmental reservoirs, and insufficient infection control were not evaluated in this study.^5,28,30–33^

Among CRE isolates, NDM, OXA-48, and coexistent NDM and OXA-48 were common carbapenemases detected, while the study did not identify isolates carrying KPC, consistent with epidemiological studies in Thailand.^34^ KPC is widely prevalent in the USA, South America, East Europe, and China but is scarcely detected in Southeast Asia.^35^ In Thailand, NDM was initially reported in 2012 and spread widely across the country.^36^ Antimicrobials against NDM-carrying CRE isolates are limited, which can cause poor treatment outcomes and increased healthcare burdens in our hospital.^37^ OXA-48 carbapenemases have weak to moderate hydrolytic activity against carbapenems; thus, OXA-48-carrying CRE can exhibit variable susceptibility patterns against these agents, as we observed.^38^

This study has several limitations. Enrollment concluded before reaching the expected sample size due to the need for additional resources and laboratory staff to continue microbiological testing. Due to the small sample size, we could not establish a strong relationship between various clinical parameters and the acquisition of CRE in hospitalized patients. Furthermore, environmental reservoirs and adherence to infection prevention and control policies may be associated factors in the carriage of CRE but were not included in our study. We did not conduct active rectal swab culture surveillance on all hospitalized patients within a single unit. As a result, we may have overlooked patients with silent CRE colonization, potentially representing another unidentified source of CRE. Some non-critically ill subjects, especially in the CRE-negative culture group, had brief hospital stays of up to 5 days. Consequently, we could not perform more extensive weekly rectal swab surveillance in these instances, which may lead to an underestimation of the prevalence of CRE acquisition. The timing of CRE infection and the colonization interval remains unclear; therefore, a long-term surveillance study in non-critically ill settings is necessary to evaluate the long-term outcomes of CRE. We used both phenotypic and genotypic testing as confirmatory methods. However, genotypic tests were unable to detect carbapenemases in 5 isolates, leaving the mechanism of resistance to carbapenem in those isolates inconclusive.

In conclusion, short-term rectal swab surveillance in non-critically ill patients who received systemic antimicrobial therapy shows a moderate increase in the prevalence of CRE acquisition. The study did not identify any independent clinical factors strongly associated with a positive CRE culture; however, factors such as the severity of illness, use of piperacillin-tazobactam, duration of antimicrobial therapy, and length of hospital stay may increase the risk of acquiring CRE. The significance of CRE surveillance through rectal swab culture in non-critically ill patients is particularly concerning, especially for those with these risk factors.
